# Supplementary material for: Public perceptions of non-adherence to pandemic protection measures by self and others: A study of COVID-19 in the United Kingdom
Source: PLoS One. 2021 Oct 28;16(10):e0258781. doi: 10.1371/journal.pone.0258781 (PMC8553167; doi:10.1371/journal.pone.0258781)
Supplement: S1 File — (DOCX) [file pone.0258781.s001.docx]

**S1 File: Focus Group schedule**

**Focus group schedule for Public Views on the Coronavirus Pandemic (PVCOVID) Study**

- Welcome the participants
- Briefly explain the aims of the research, who is conducting it, and how it is funded, and the fact it has ethical approval.
- Remind the participants:
  - They can stop participating at any point, particularly if they feel uncomfortable
  - That all data will be used completely anonymously.
  - That data will be recorded then transcribed and stored securely for a period of time.

**General views on social distancing**

- In what ways has the COVID pandemic affected your daily lives?
- What is your understanding of what ‘social distancing’ means?
- How closely have you been sticking to the government’s social distancing requirements?
  - Probe if needed (e.g. keeping 2 meters distance, only meeting or mixing with others as and where permitted, complying with stay-at-home orders (lockdowns) when needed)
- Do you think the government’s measures have been too harsh, about right or too slow or lenient? Why do you think this?
- Have there been any challenges or difficulties with meeting requirements and recommendations on social distancing?

**Impacts of the pandemic**

- Is social distancing affecting your:
  - Physical health
  - Mental or emotional health
  - Quality of life (general challenges?)
- Do you think that longer term, continued social distancing and other coronavirus policies could have an impact on your physical or mental wellbeing?
- Is there anything you feel like you are losing or have lost as a result of social distancing?

**Looking ahead**

- How long do you think social distancing is going to last? How long do you plan on doing it for?
  - Do you think it will be easy or difficult to re-adjust when current social distancing measures are relaxed or removed?
- How do you think you are going to act when the current social distancing measures are relaxed or removed?

**Public understanding of measures**

- Do you think the government has done a good job communicating to the public? - has the social distancing guidance been clear?
  - Probe, if relevant: What are some of the things that the government might do to improve their communication to the public?
- What are your views on whether the public should be given more say in Covid policy?
- Can the government be trusted on this matter?
- Where do you get your information about Covid and Covid measures from?
- How concerned are you about coronavirus?

**Any other thoughts?**
